# Design and rationale for the WARFA trial: a randomized controlled cross-over trial testing the therapeutic equivalence of branded and generic warfarin in atrial fibrillation patients in Brazil

**DOI:** 10.1186/s12872-017-0584-4

**Published:** 2017-06-07

**Authors:** Carolina Gomes Freitas, Michael Walsh, Álvaro Nagib Atallah

**Affiliations:** 1 Department of Medicine, Universidade Federal de São Paulo (UNIFESP), CEPATIS, Rua Botucatu, 740 - 3o andar, São Paulo/SP, CEP 04023-900 Brazil; 2 Discipline of Emergency Medicine and Evidence-Based Medicine, Universidade Federal de Sao Paulo (UNIFESP), CEPATIS, Rua Botucatu, 740 - 3o andar, São Paulo/SP, CEP 04023-900 Brazil; 3 Cochrane Brazil, Rua Borges Lagoa, 564 - cj 63, São Paulo/SP, CEP 04038-000 Brazil; 4 Department of Medicine (Nephrology) and Health Research Methods, Evidence and Impact, McMaster University, Hamilton, Canada; 5 Population Health Research Institute, McMaster University/Hamilton Health Sciences, Hamilton, Canada

**Keywords:** Warfarin, Generic drug, Generic drugs, Brazil, Statistical analysis plan, Crossover, Randomized crossover trial, Randomized controlled trial crossover design, Therapeutic equivalence, Atrial fibrillation

## Abstract

**Background:**

Warfarin is a commonly used anticoagulant. Whether a given dose of the different formulations of Brazilian warfarin will result in the same effect on the international normalized ratio (INR) is uncertain. The aim of the WARFA trial is to determine whether the branded and two generic warfarins available in Brazil differ in their effect on the INR.

**Methods:**

WARFA is a cross-over RCT comparing three warfarins. The formulations tested are the branded Marevan® (Uniao Quimica/Farmoquimica) and two generic warfarin (manufactured respectively by Uniao Quimica Farmaceutica Nacional and Laboratorio Teuto Brasileiro). All of them were manufactured in Brazil, are available in all settings of the Brazilian healthcare system and were purchased from retail drugstores. Eligible participants had atrial fibrillation or flutter, had been using warfarin for at least 2 months with a therapeutic range of 2.0–3.0 and had low variability in INR results during the 1st period of the trial. Our primary outcome, for which we have an equality hypothesis, is the difference between warfarins in the mean absolute difference between two INR results, obtained after three and 4 weeks with each drug. Our secondary outcomes, that will be tested for inequality (except for the mean INR, which will be tested for equality), include the difference in the warfarin dose, and time in therapeutic range. Clinical events and adherence were also recorded and will be reported.

**Discussion:**

To our knowledge, WARFA will be the first comparison of the more readily applicable INR results between branded and generic warfarins in Brazil. WARFA is important because warfarins are commonly switched between in the course of a chronic treatment in Brazil. Final results of WARFA are expected in May 2017.

**Trial registration:**

ClinicalTrials.gov NCT02017197 . Registered 11 December 2013.

**Electronic supplementary material:**

The online version of this article (doi:10.1186/s12872-017-0584-4) contains supplementary material, which is available to authorized users.

## Background

Novel drugs’ efficacy and safety must be proven by randomized clinical trials (RCTs). Generic drugs, however, follow different regulations and instead, are required to demonstrate their similarity to the branded product, already proven efficacious and safe [1].

There are different criteria on how to prove similarity depending on the pharmaceutical product and on the active pharmaceutical ingredient. Generally, for generic drugs in solid oral dosage formulations (e.g. tablets), bioequivalence studies are a requisite [2–4]. These studies assess whether the amount of drug absorbed and the rate of absorption between the generic and the branded drug do not show differences greater than prespecified limits [2]. In Brazil all bioequivalence claims must still show evidence that the 90% confidence interval (CI) of the difference or of the ratio between the means of the generic and the branded drug comply with the limits of +20% and −20% of the branded drug mean^1^ [5, 6], whereas a recent guideline set narrower limits for low therapeutic index drugs [2].

There is debate on whether generic warfarin is really equivalent to the branded drug [7–13]: first because this is a drug with a narrow therapeutic index [14, 15], whose effects must be strictly controlled in order to balance benefits and risk; moreover, because generic drugs’ effects are not routinely assessed in randomized controlled trials (only surrogate bioequivalence outcomes). Consequently, potentially wide equivalence intervals for generic drugs may result in excessive variation in the effects of warfarin that lead to suboptimal anticoagulation or the need for additional monitoring.

This question has been addressed before with RCTs [9–13] but, to our knowledge, never with drugs manufactured in Brazil. Therefore, they may not be applicable to the Brazilian setting, since bioequivalence, which relies on bioavailability, is highly dependent on the formulation and manufacturing of the drug. Also, in Brazil, an even more relevant question is whether there is equivalence also within the generics since it is usual for patients in their chronic treatment to receive, from hospitals (either public or private) or from other public healthcare settings, the same drug manufactured by different laboratories.

The aim of the WARFA trial is to compare the branded (Marevan^®^) with two generic warfarins available in Brazil for therapeutic equivalence. This paper describes the design of the trial and our analysis plan.

## Methods

### Study design and setting

WARFA is a single-centre cross-over RCT. The trial features 3-drugs, 4-periods (1 month each), 6-sequences and is depicted in Table 1. The allocation ratio to each sequence is 1:1:1:1:1:1. A populated SPIRIT (Standard Protocol Items: Recommendations For Interventional Trials) checklist is provided as the Additional file 1.

**Table 1:** Schema for the cross-over design of the study

Periods^a^	Period 1	Period 2	Period 3	Period 4
Sequences				
AABC	A	A	B	C
BBAC	B	B	A	C
BBCA	B	B	C	A
AACB	A	A	C	B
CCAB	C	C	A	B
CCBA	C	C	B	A

*B* Branded warfarin, *A* and *C*, Generic formulations of warfarin, each from a different manufacturer

^a^Each period is one-month long

Patient recruitment started in August 2014 and was finished by March 2016; follow-up was concluded by August 2016. Patients were selected from the outpatient anticoagulation clinic at the Hospital Sao Paulo, a public university hospital in the city of Sao Paulo (Sao Paulo State, Brazil).

### Ethics approval, consent to participate and trial registration

Ethical approval has been received from the research ethics committee at the Federal University of São Paulo (CAAE 20758713.3.0000.5505). All patients signed the consent form prior to inclusion in the trial. The trial was registered at ClinicalTrials.gov (NCT02017197) on 11 Dec 2013, before the inclusion of the first patient.

### Study population

The eligibility criteria were designed to select a subset of homogeneous patients that received warfarin and for whom warfarin is indicated and safe. Eligible patients had to meet all the following criteria: age ≥ 18 years; atrial fibrillation (AF) (permanent, persistent or paroxysmal, but not transient AF) diagnosed by physicians in the absence of a mechanical valve and documented with electrocardiogram results; CHA2DS2VASc score ≥ 1 [16, 17]; on warfarin for at least 2 months prior to randomisation; able to provide written informed consent to participate.

Patients with any of the following exclusion criteria were not eligible: patients for whom warfarin was contraindicated (e.g. use of another anticoagulant, recent significant bleeding, known sensitivity to warfarin); pregnant, breastfeeding or women of childbearing potential; severe thrombocytopenia (<40,000/μL); advanced hepatic (i.e. liver cirrhosis) or renal failure (eGFR <15 mL/min/1.73m^2^); history of major bleedings due to congenital deficiency of coagulation factors; participating in another clinical trial; patients starting chronic treatment with drugs that have moderate and/or major interactions that might affect the INR, and/or the warfarin dose, and/or the risk of bleeding when used concurrent with warfarin (according to the online database Micromedex® 2.0 [18]).

After randomization, only patients with low variability of their INR results during the first 4 week period continued in the study. Patients were kept in the study if at least one of the three (including the baseline) INR results were between 2.0 and 3.0 and the difference of INR results at the 3th and 4th week ≤ ±0.8. This decision was made to reduce the amount of variability in INR values created by non-drug sources (e.g. patients with variable vitamin K intake) and therefore increase the probability of detecting true between drug differences in INR.

### Interventions and controls

Patients received three different formulations of warfarin sodium tablets, one in each period, according to the sequences to what they were randomized. The branded one was Marevan®, from Uniao Quimica/Farmoquimica, and the other two were generics manufactured respectively by Uniao Quimica Farmaceutica Nacional and Laboratorio Teuto Brasileiro [19]. All of them were manufactured in Brazil and are available in all settings of the Brazilian healthcare system (both public and private). The drugs used in the trial were purchased from a retail drugstore, not directly from the manufacturers, to enhance the comparability of the trial results to “real-world” conditions.

Patients were oriented to take the warfarin 5 mg tablets (the most widely available dose form) once a day, at the same time each day. If needed for their daily dose, patients would split the tablets to receive 2.5 mg dose increments/decrements. Study visits were planned in the 3rd and 4th week after starting every different warfarin; at each visit, if necessary, warfarin dosage was adjusted to keep the INR results within the therapeutic range of 2.0 to 3.0.

Adjustment of the warfarin dose followed an adapted version of the ENGAGE AF-TIMI 48 trial protocol for dose adjustment (Additional file 2) [20]. The criteria described in the guideline was applied if at least two consecutive INR results were out of range. Exceptions were when INR results were <1.5 or else >3.5: in these cases, a single INR result out of range could motivate adding extra doses or else suspending subsequent warfarin doses. When INRs were out of range extra visits could be scheduled for monitoring INR results and adjusting warfarin dosage.

Concomitant medication was allowed. All patients were instructed to: take their other drugs (for other chronic or acute conditions) as instructed by their physicians; avoid self-medication (patients were advised to avoid nonsteroidal anti-inflammatory drugs (NSAIDs) and use the over-the-counter acetaminophen or dypirone if needed for minor occasional pain, due to their low potential for interaction with warfarin); and report all new drugs (i.e., a drug that he/she had not been taking since baseline) to the investigator.

At the study visits the investigator inquired and registered concomitant medications. Before the analysis, INRs will be classified as valid or invalid. We will consider INRs valid if the measurements due were taken when the patient was using the drug to which he/she was assigned without interference of current diarrhoea. We will consider INR measurements as invalid if they were collected: 1) when a new drug with major or moderate interactions was used in the last 24 h along with warfarin (for drugs with moderate interactions, they also had to be used on a continuous basis or at least more than once in the last 24 h); 2) after suspension of a drug with moderate or major interactions previously taken since baseline. We only considered interactions that, according to Micromedex^®^ 2.0 [18], might alter the INR, and/or the warfarin dose, and/or the risk of bleeding.

Treatment with warfarin had to be suspended in the following situations: for a limited and short time if patients had to be submitted to specific surgical interventions, as recommended by the American College of Chest Physicians [21]; or else in the case of major bleeding. In the latter, treatment was not resumed until the source of the bleeding was identified and we were sure that the patient would be more benefitted than harmed by the treatment.

Compliance was measured by pill count at the end of each period. Though INR figures as an outcome, it was also used, coupled with pill count, as an ancillary measure of adherence. Feedback was given to patients as a way of ensuring adherence.

### Outcomes

For planning the outcomes, our concern was to address two possible situations: 1) tablets within a manufacturer could have varying doses or bioavailabilities due to inadequate quality control; 2) between manufacturers, tablets could have consistently dissimilar bioavailability (e.g. one formulation with a high bioavailability in comparison to the others). Both situations could pose risks to the patient by placing them out of the therapeutic range [22] and/or incur in the need for more consultations for dose adjustment.

To reflect these potential situations, our primary outcome is the difference in Δ INR. The Δ INR is the absolute difference between the two INR results obtained by the patient with the same drug formulation at each period (Table 2). This measurement is intended to address variation in the INR results. We have an equivalence hypothesis and we will accept the warfarins as therapeutic equivalent if the 95% CI (lower and upper bounds) of the difference between the mean Δ INR of the formulations is within −0.49 and +0.49. The bounds for the equivalence test were determined by consensus with a cardiologist experienced in warfarin management as the minimal important difference in the INR results that would support a dose change.

**Table 2:** Schedule of enrolment, interventions, and assessments of the WARFA trial

	STUDY FOLLOW-UP								
	Enrolment/ Randomization	1st Month/ Period		2nd Month/ Period		3rd Month/ Period		4th Month/ Period	
WEEK	0	3	4	7	8	11	12	15	16
ENROLMENT									
Eligibility screen	X								
Informed consent	X								
Allocation	X								
INTERVENTIONS									
Assessment of INR variability			X						
Switch of warfarin formulation	X				X		X		
ASSESSMENTS									
Assessment of patient stability in warfarin treatment			X						
INR	X	X	X	X	X	X	X	X	X
PT	X	X	X	X	X	X	X	X	X
Warfarin dosage	X	X	X	X	X	X	X	X	X
Stroke	X	X	X	X	X	X	X	X	X
Major Bleeding	X	X	X	X	X	X	X	X	X
Death	X	X	X	X	X	X	X	X	X
Systemic thromboembolism	X	X	X	X	X	X	X	X	X
Minor bleeding	X	X	X	X	X	X	X	X	X
Adherence			X		X		X		X

This equivalence interval was intended originally for the outcome of the difference between mean INRs, our original primary outcome, but we thought it was suitable for this new primary outcome as well. We made this change to the protocol because we felt that, for being an average between only two measurements, the mean INR would not be sensitive enough to identify important variations in INR results between treatments. Thus we replaced the mean INR (that turned to a secondary outcome) by the Δ INR as the primary outcome.

The difference between mean INRs for each formulation is one of the secondary outcomes for which we also have an equivalence hypothesis using exactly the same criteria (equivalence interval within −0.49 and +0.49 and decision based on the 95% CI for the difference of the mean INRs fitting entirely into it). Differently from the primary outcome, this measurement is intended to address systematically lower or higher mean INR results.

For the other secondary outcomes we have inequality hypothesis. They are: the difference between mean warfarin dosage needed, the difference between Δ warfarin dosage needed (the absolute difference between the two weekly warfarin dosages needed by the patient with the same drug formulation at each period) and the difference between mean time in therapeutic range (TTR). Clinical events (thromboembolic, bleeding events, deaths and other adverse effects) were also recorded and we will present their frequencies, but we will not conduct hypothesis tests because these were not the main outcomes in this trial and we were very likely underpowered for detecting differences between treatments; also, the cross-over design was not suitable for assessing these kind of outcomes since most of them would lead to suspension of the warfarin treatment for indeterminate time, making it impossible for the patient to be exposed to the next interventions in the sequence. The adherence with treatment was also measured and we will present it in a dichotomous way (e.g. patients had at least 80% adherence).

A secondary outcome that was planned at the beginning but we have discarded for the final analysis is the difference between mean prothrombin times (PTs). The PT is the basis for the INR, which was created to address the problem of comparability of laboratory results [23] and it is a measure widely used nowadays. Thus we decided that analyzing the PT in addition to the INR would be unnecessary since it would convey the same information as the INR.

INR, warfarin dosage, and clinical events will be measured at the study visits in the 3rd and 4th week of each period. The outcomes of Δ INR, mean INR, Δ dosage, mean dosage and mean TTR will also be based on these measurements. Adherence will be measured in the 4th week of each period.

The controls for each intervention will vary according to the comparison being made: when comparing the generic formulations to the branded we will use the latter as the control; when comparing generics to each other, we will use always the same generic formulation as the control for the other.

### Randomization and blinding

We applied block randomization (fixed block size of 6). Patients were randomly allocated to the sequences of treatment by numbered, opaque, sealed envelopes that would designate patients to sequences identified with letters from A to F. The envelopes were prepared, randomly ordered and then numbered by the PI according to the SNOSE method [24]. This randomization method allows the concealment of the allocation sequence even from the person performing the steps for randomization, once that the sequence results from the shuffling and numbering of the sealed and opaque envelopes (i.e., there is no register of the randomization sequence).

Researchers external to the study (BR and MFST) put the drugs in opaque plastic mailing bags of identical appearance, and then sealed and identified them only by letters corresponding to allocation sequences and the study periods (e.g. A, 2nd month), allowing blinding of personnel involved in the treatment of the patients (the PI and the cardiologists) and in the collection of data (the PI). These external researchers are the only ones who know the code of letters for the sequences of treatment. Copy of this allocation list is stored in an opaque and sealed envelope that will be opened only at the end of the study, at the time of statistical analysis by the PI. With this process, besides generating the allocation sequence, the PI was be able to enrol participants, assign them to the intervention and also follow them up without compromising the allocation concealment and the blinding/masking.

Since every patient would be taking warfarin, we decided that blinding should be kept throughout the trial and unblinding would not be permitted. In the case of any emergency patients would be treated accordingly, knowing that they were taking warfarin.

Patients, however, were not blinded to treatments. The warfarin tablets from different manufacturers were different in their appearance, but we decided to keep them in their original packaging (we just added the external opaque bag to blind personnel) in order to avoid interfering with their stability or else with their bioavailability.

### Carry-over effect

Since we could not take the patients off warfarin for a complete washout for ethical reasons, the INR measurements planned were taken only after 3 weeks after the switch of formulations (Table 2) as a way of ensuring that the effects of drugs administered in the previous period would not contaminate the outcomes. This period is superior to 5 half-lives (warfarin’s half-life: 25 to 60 h [25]), time that is considered enough in pharmacokinetics for clearance of drugs from the body [26].

In addition to addressing carry-over in the design of the trial, we will also use the approach described by Jones and Kenward to test for carry-over effects in the statistical analysis stage [27].

### Sample size

Sample size calculation was based on the outcome of the mean INR, which, as described before, was our previous primary outcome. It was intended to detect a clinically significant difference of 0.49 in the mean INR, assuming a significance level of 5 and a 90% power.

Data were inputted in the formula presented as a statistical method for a quantitative outcome [28]. This formula is intended for a parallel trial (independent groups) and we expected it to result in a super estimation of the sample size in this trial due to its cross-over design (i.e. dependent observations) [29].

Standard deviation for the INR inputted in the formula was 0.34, which was the higher standard deviation shown for the mean INR of a warfarin formulation in a previous trial (calculated from a standard error reported) [9]. These assumptions resulted in a sample size of 11 patients in each group. Thus, if the trial was parallel, we would need 33 patients for detecting this difference. Since the trial was a cross-over, we would then probably need a smaller sample size.

First, we aimed to recruit at least 33 patients completing the trial. Due to the high rate of subjects not meeting the INR stability criteria in the 1st period we aimed to recruit 100 patients.

### Statistical analysis

We did not plan or conduct any interim analyses. We will analyze data with the software STATA/IC 14.0 for Windows. Data will be modelled using a multilevel mixed-effects linear regression with random intercepts. We will include terms for: the sequence of administration of treatments [30]; the time-periods that the treatments were administered [30, 31]; the treatment; the carry-over effect and also for the patient [31]. All the effects will be fixed, except for the patient that will be a random intercept. We will not use baseline measures as covariates since we did not take patients out of the drug (wash out) and thus baseline measures are the result of different warfarin formulations that patients were taking before the trial. For all analysis of continuous outcomes we will present the mean differences and confidence intervals so that it can be shown whether the results are conclusive or not [32].

For assessing the effects of the other terms besides the treatment on the outcome, we will use a two-staged procedure [33], adding these effects to the model and testing them for significance: if any of them is significant at the 5% level, treatment effects are deemed not equal across periods and thus we will not pool them together in a cross-over fashion. Only data from the first treatment period, in a parallel-like fashion, will be used then [30]. On the other hand, if none of them are significant, these variables will be dropped from the model and data from the other periods (2nd, 3rd and 4th months) will also be considered in the analysis. In the latter, all the following planned analysis for the outcomes will be performed.

We will not impute missing outcome data. Because the potential direction and magnitude of biases from missing data are unpredictable, we decided to analyse three populations based on their pattern of missing outcome data. To be maximally conservative, if any of these analyses demonstrate non-equivalence between warfarin formulations, we will consider that formulation non-equivalent (i.e. all three analytic populations are considered equally). We will present patient baseline characteristics for each analytic population.

The first analytic population is referred to as “*Complete cases*”. This encompasses data from patients that had at least one valid INR in *every* treatment period (or ΔINR, for which at least 2 valid INRs would be necessary).

Another population for analysis is called “*First treatment period group*” and it is comprised of *only* the data of the 1st period for patients with at least one valid INR or ΔINR in this same period. This population retains the randomization balance. Including also the data from patients with high variability in the analysis and considering this data as a parallel comparison between the warfarin formulations will help us assess whether the same conclusions as for the complete cases hold, even if our initial assumption about confounders in patients with high INR variability does not.

The last population for analysis is named “*modified ITT*” and it is composed of patients with at least one valid INR or ΔINR in *any* time period. This would include all valid data from all patients randomized. Using the linear mixed model for the cross-over we are able to include even the incomplete data from patients that did not complete treatment sequences and account for them in the analysis. However, since they do not have as much information as the other patients (i.e. there are no measurements in some periods), they will be consequently accounted with less weight than the patients that actually completed the trial.

## Discussion

The WARFA trial addresses the therapeutic equivalence issue of generic warfarin in Brazil in a novel way. Besides assessing therapeutic equivalence (not bioequivalence) between the effect of generics and the branded drug, an unmet need in Brazil, we will also focus on comparing among generic drugs. Despite not devised to be therapeutically equivalent to each other, generic drugs from different manufacturers are commonly switched in the course of a chronic treatment in Brazil, and thus it is desirable to evaluate their suitability for this purpose. Our question may initially be of interest for Brazilians but warfarin is still used worldwide and we believe that the switch between many different brands is not a situation restricted to Brazil. Therefore, we consider that our methods of approaching this issue will be of interest of a wider audience.

Detecting small differences within generic and branded drugs motivated us to use a cross-over design. This design focuses on the analysis of what is called *within-patients* differences: the difference between effects of treatments observed in the same individual, which acts as its own control. The within-patients estimates of treatment effects ensure balance of confounding factors inherent to patients [34].

Nevertheless, cross-over designs also presents drawbacks: the sequences to which patients are randomized differ with respect to their recent exposure to other potentially effective treatments (i.e. the treatment applied in the previous period). Thus, the trial design alone does not guarantee comparability between treatments, because this depends also on the treatment effects being confined to the period of their administration and follow-up. When this condition does not hold and the effect of one treatment contaminates the treatment in the following period, it causes a carry-over effect [34]. In our trial, we planned to address carry-over effects in the design (the timing of the outcome measures) and also in the analysis of the data (testing for carry-over effects and analysing data accordingly).

Another challenge of cross-over trials is dealing with missing data. In conventional analysis, in which subjects are considered fixed effects (e.g. T-test), a value missing for a patient in one period of the trial compromises all the information of that subject. Since there is no within-patient difference for that individual, the incomplete information cannot be used for estimation of the treatment effects and is thus discarded. Conversely, mixed models that assume random subjects have the advantage of being able to use the available information even when data are missing in a cross-over design [31, 34]. For dealing with the missing data issue, not only we are using linear mixed models in the analysis, but we also planned to use different populations, taking advantage from the 1st period, in order to check whether our assumptions change the conclusions of the trial.

In this article we described some changes from the original protocol in the outcomes. We also had to change the eligibility criteria in order to recruit more patients. At first, only nonvalvular AF patients were eligible to the WARFA trial. However, we decided to include also atrial flutter (AFL) patients and AF/AFL valvular patients without mechanical prosthetic valves, i.e. AF/AFL patients with rheumatic disease or mitral stenosis, because they all receive anticoagulation with warfarin in the same therapeutic range as the nonvalvar AF patients [35]. Since we expect the same effect of warfarin in preventing thromboembolic events in all these subjects, we do not expect that this decision will bias our estimates [36]. All the modifications have been described, were planned up to the statistical analysis plan stage, i.e. before data analysis and also before unblinding, and we do not expect them to bias our estimates.

Unfortunately, due to time and resources restrictions, we were not able to couple the WARFA trial with bioequivalence studies, that would help us compare bioavalabilities to warfarin results. However, we focused on the more readily applicable question, of whether there is actual therapeutic equivalence between the formulations available in Brazil.

### Trial status

Patient recruitment and follow-up are already completed. At the time of submission, we finished the statistical analysis plan and are working on study close out. Final results are expected by May 2017.

## Additional files

Additional file 1: Populated SPIRIT (Standard Protocol Items: Recommendations For Interventional Trials) checklist for the WARFA trial. Populated SPIRIT checklist for the WARFA trial. (DOCX 54 kb)

Additional file 2: Guidance for warfarin dose adjustment applied in the WARFA trial. Chart showing the protocol applied in the WARFA trial for adjustment of warfarin dose. (DOCX 12 kb)

## Abbreviations

AF: Atrial fibrillation
AFL: Atrial flutter
CI: Confidence interval
eGFR: Estimated glomerular filtration rate
INR: International normalized ratio
NSAIDs: Nonsteroidal anti-inflammatory drugs
PI: Principal investigator
PT: Prothrombin time
RCT: Randomized controlled trial
TTR: Time in therapeutic range
UTN: Universal Trial Number

## Footnotes

1 All the limits follow the same ±20% rule. However, the actual values for the limits differ according to the scale used (arithmetic or logarithmic) and the kind of comparison being made (difference or ratio) [6].
