# Supplementary material for: Design and rationale for the WARFA trial: a randomized controlled cross-over trial testing the therapeutic equivalence of branded and generic warfarin in atrial fibrillation patients in Brazil
Source: BMC Cardiovasc Disord. 2017 Jun 7;17:148. doi: 10.1186/s12872-017-0584-4 (PMC5463418; doi:10.1186/s12872-017-0584-4)
Supplement: Supplementary file 2 — Guidance for warfarin dose adjustment applied in the WARFA trial. Chart showing the protocol applied in the WARFA trial for adjustment of warfarin dose. (DOCX 12 kb) [file 12872_2017_584_MOESM2_ESM.docx]

| Guidance for warfarin dose adjustment applied in the WARFA trial. | |
| --- | --- |
| **INR** | **Suggested warfarin dose adjustment** |
| < 1.5 | Increase weekly dose by 10% to 20%  Consider giving an extra dose  Retest INR in 4 to 8 days or per Investigator discretion |
| 1.5 to < 2 | Increase weekly dose by 5% to 10%  Retest INR in 7 to 14 days or per Investigator discretion |
| 2.0 to 3.0 | No change |
| > 3.0 to 3.5 | Decrease weekly dose by 0% to 20%  Retest INR per Investigator discretion |
| > 3.5 to 4.0 | Withhold 0 to 1 dose  and/or  Decrease weekly dose by 0% to 20%  Retest INR per Investigator discretion |
| > 4.0 but < 5.0 | Withhold 1 to 2 doses  and  Decrease weekly dose by 0% to 20%  and  Retest INR in 3 to 7 days or per Investigator discretion |
| 5.0 to < 9.0  without significant bleeding | Withhold 1 to 2 doses  Retest INR in 1 to 2 days or per Investigator discretion  Resume dosing once INR < 3.0, but with weekly dose decreased by 5% to 20%  If the subject needs urgent surgery, then the subject should receive Fresh Frozen Plasma |
| > 9.0  without significant bleeding | Withhold study drug  Give Vitamin K (single 2.5 to 5 mg oral dose)  Repeat INR test daily until INR < 5.0  If INR remains too high, more Vitamin K doses can be considered.  Resume dosing once INR < 3.0, but with weekly dose decreased by 10% to 20%.  If the subject needs urgent surgery, then the subject should receive Fresh Frozen Plasma |
| Adapted from the protocol for the ENGAGE AF-TIMI 48 trial [24]. | |
